# Similia Similibus Curantur

**Published:** 1888-03

**Authors:** 


Similia Similibus Curantur. By Charles S. Mack, M. D. Price, 40 cents. Otis Clapp & Son, Boston and Providence. 1888.

To the impartial observer it would seem curious that one should undertake to write on a practical subject with which he confesses he has had very limited experience! Yet this is just what Dr. Mack does in this essay upon "Similia Similibus Curantur."

Like many others, Dr. Mack has not made himself familiar with the true principles of Homœopathy; had he done so he would never have written this little essay, nor would he have been in doubt in distinguishing between accidental recoveries and cures. Hahnemann long ago pointed out the distinguishing features.

